# Feasibility of replacing ^99m^Tc‐DTPA GFR measurements with eGFR from cystatin C in individuals with spinal cord injuries

**DOI:** 10.14814/phy2.70315

**Published:** 2025-05-12

**Authors:** Tatiana Kristensen, Peter S. Oturai, Bryan T. Haddock, Fin Biering‐Sørensen, Christina Kruuse, Ulrik B. Andersen

**Affiliations:** ^1^ Department of Clinical Physiology and Nuclear Medicine Copenhagen University Hospital ‐ Rigshospitalet; Glostrup Copenhagen Denmark; ^2^ Department of Brain and Spinal Cord Injury, Neuroscience Center Copenhagen University Hospital ‐ Rigshospitalet; Glostrup Copenhagen Denmark; ^3^ Institute of Clinical Medicine, University of Copenhagen Copenhagen Denmark

**Keywords:** cystatin C, DTPA‐clearance, eGFR, GFR, kidney dysfunction, spinal cord injury

## Abstract

In individuals with spinal cord injury (SCI) and neurogenic bladder dysfunction, guidelines recommend regular monitoring of kidney function by measuring the glomerular filtration rate using an externally administered filtration markers such as 99mTc‐DTPA, since creatinine‐based eGFR models are inaccurate due to lower muscle mass in these individuals. To examine the feasibility of substituting GFR measurements with eGFR based on s‐cystatin C, simultaneous 99mTc‐DTPA clearance (mGFR) and cystatin C‐based clearance (eGFRcys) measures were evaluated in 248 individuals with SCI. In a subgroup of 26 participants, the test–retest variability of eGFRcys was assessed. Finally, long‐term (1–3 years) repeatability of simultaneously measured mGFR and eGFRcys was evaluated in 40 individuals. We could demonstrate a very good correlation between mGFR and eGFRcys, with an intraclass correlation (ICC) of 0.92, a very good test–retest variation of eGFRcys (ICC: 0.98) and a very good long‐term repeatability of eGFRcys and mGFR (ICC 0.92 and 0.94, respectively). We conclude that in individuals with SCI, eGFR calculated from a single sample of cystatin C can replace measurements of GFR using an externally administered substance. Using a fixed normal limit rather than an age‐corrected normal material for p‐cystatin C or eGFRCYS will misclassify many individuals as having chronic kidney disease.

## 1 INTRODUCTION

Individuals with spinal cord injury (SCI) and neurogenic bladder dysfunction (Hamid et al., 2018) are at increased risk of developing renal failure (Schmitt et al., 1991; Wall et al., 1999), due to incontinence, reflux, urinary tract infections, and nephrolithiasis. The risk of developing renal failure is approximately sevenfold higher in individuals with SCI compared to age‐matched healthy controls. Dilation of the urinary tract and obstructing kidney stones that require removal are risk indicators predicting deterioration of kidney function (Elmelund et al., 2016; Lawrenson et al., 2001). Therefore, according to international guidelines, these individuals should have a kidney function test and imaging of the urinary tract at regular intervals (Abrams et al., 2008; Ginsberg et al., 2021).

The glomerular filtration rate (GFR) is a useful measure of kidney function. GFR can be estimated from the serum level of endogenous markers such as creatinine and cystatin C, or it can be measured by injecting an exogenous marker such as inulin or radiolabeled diethylenetriaminepentaacetic acid (DTPA) (Levey & Inker, 2017; Stevens et al., 2008, 2013). In routine practice, s‐creatinine measurements are most used due to better availability and lower costs (Levey & Inker, 2017; Perrone et al., 1992). However, creatinine levels in the blood vary according to gender, race, intake of creatinine in the food, and individual muscle mass. To compensate for these variations, equations to estimate GFR from s‐creatinine are routinely used to assess renal function. The most commonly used are the Chronic Kidney Disease Epidemiology Collaboration (CKD‐EPI) equations (Inker et al., 2012, 2021; Levey et al., 2009).

In individuals with SCI, due to the reduced muscle mass following SCI with subsequent muscle inactivity, even the CKD‐EPI equations are inaccurate and overestimate the true creatinine clearance (Erlandsen et al., 2012; Goto et al., 2018). For this reason, it is recommended that renal function is estimated from the clearance of an externally administered filtration marker such as ^99m^Tc–diethylenetriaminepentaacetic acid (^99m^Tc‐DTPA). ^99m^Tc‐DTPA is primarily excreted by glomerular filtration and is an accurate measure of GFR (Vidal‐Petiot et al., 2021). However, measuring GFR using ^99m^Tc‐DTPA is more time‐consuming, expensive, and labor‐intensive compared to serum creatinine‐determined GFR.

Cystatin C has been proposed as an alternative marker for estimating GFR (Shlipak et al., 2013). Cystatin C is an endogenous protein filtered by the glomeruli and in turn reabsorbed and catabolized by the tubular epithelial cells with only small amounts excreted in the urine. The production rate of cystatin C is relatively constant and unaffected by age, gender, race, meat intake, or muscle mass (Inker et al., 2012, 2021). The interindividual variation of s‐cystatin C is much smaller than for s‐creatinine. Cystatin C can detect renal deterioration in individuals with SCI (Erlandsen et al., 2012; Mingat et al., 2013; Thomassen et al., 2002), and CKD‐EPI equations have been developed to estimate GFR from s‐cystatin C alone or a combination of s‐cystatin C and s‐creatinine (Inker et al., 2012, 2021; Levey et al., 2009).

This retrospective study aimed to establish if s‐cystatin C measurements could replace GFR measurements using an external filtration marker. Measures of estimated GFR from s‐cystatin C levels (eGFR_CYS_) were compared to simultaneous GFR measures using the gold standard ^99m^Tc‐DTPA clearance (mGFR) in individuals with SCI. Furthermore, the test–retest variability of eGFR_CYS_ in individuals who had repeated sampling within 3 months was established. Finally, simultaneous measurements of eGFR_CYS_ and mGFR taken within a 2–3 years interval in a subset of individuals were used to compare long‐term repeatability of the methods.

## 2 RESULTS

A total of 248 individuals with SCI (153 men and 95 women) aged 20–89 years participated in the study and completed all the measurements. Characteristics of the participants are listed in Table 1. There were no significant gender‐related differences for age, height, body surface area (BSA), body mass index (BMI), mGFR, s‐cystatin C, eGFR_CYS_, or eGFR_CYS‐CREA_, but weight, s‐creatinine, and eGFR_CREA_ were higher in males than in females.

**TABLE 1:** Characteristics of the study cohort displayed as median (interquartile range, separated by;) or mean ± standard deviation.

	Men	Women	All participants
	(*N* = 153)	(*N* = 95)	(*N* = 248)
Age (years)	59.3 (47.5; 71.7)	62.6 (48.1; 75.6)	60.9 (47.6; 73.3)
Weight (kg)	82.0* ± 17.8	74.2* ± 16.8	79.0 ± 17.8
Height (cm)	178 ± 10	165 ± 9	173 ± 11
Body mass index (kg/m^2^)	25.4 (22.6; 29.3)	25.4 (22.7; 32.8)	25.4 (22.7; 30.2)
Body surface area (m^2^)	1.99 ± 0.23	1.80 ± 0.20	1.92 ± 0.24
GFR (DTPA clearance) (mL/min/1.73m^2^)	82.4 ± 24.1	78.1 ± 23.9	80.7 ± 24.0
S‐cystatin C (mg/L)	1.14 ± 0.53	1.10 ± 0.36	1.13 ± 0.47
S‐creatinine (mg/dL)	0.76* ± 0.36	0.62* ± 0.17	0.71 ± 0.31
eGFR_CYS_ (CKD‐EPI) (mL/min/1.73m^2^)	75.9 ± 25.1	72.0 ± 26.7	74.4 ± 25.7
eGFR_CREA_ (CKD‐EPI) (mL/min/1.73m^2^)	104* ± 26.9	97* ± 21.3	101 ± 25.0
eGFR_CYS‐CREA_ (CKD‐EPI) (mL/min/1.73m^2^)	88.5 ± 25.1	84.2 ± 25.7	86.9 ± 25.4

Abbreviations: GFR (DTPA clearance): Radiolabeled diethylenetriaminepentaacetic acid clearance. eGFR_(NN)_ (CKD‐EPI): estimated glomerular filtration rate calculated using the Chronic Kidney Disease Epidemiology Collaboration equation. cys: cystatin C, crea: creatinine.

* *p* < 0.05.

Most participants had normal relative renal function (Table 2).

**TABLE 2:** Stages of relative renal function, categorized as suggested by Brochner‐Mortensen J et al. (with the delimitations expressed as percent of corresponding age‐ and gender‐dependent normal mean GFR) (Brochner‐Mortensen et al., 1977).

Stage of relative renal function	Number of participants	Percent of participants
Normal (>75%)	198	79.8%
Moderately decreased (75%–52%)	42	16.9%
Considerably decreased (51%–28%)	5	2.0%
Severely decreased (<28%)	3	1.2%

**FIGURE 1:**
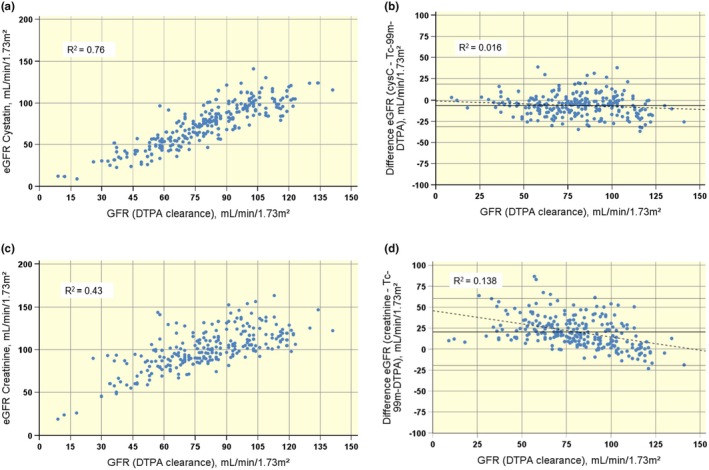
Correlation plots and Bland–Altman plots of eGFR_CYS_ and eGFR_CREA_ versus mGFR. Identity and Bland Altman plots (a and b) of the close relationship between mGFR and eGFR_CYS_, with minimal bias. In comparison, a significant bias and poorer correlation are evident in similar plots comparing mGFR and eGFR_CREA_ (c and d). Top and bottom reference lines indicate limits of agreement (narrow solid line). The thin dashed line represents the regression line. cysC, cystatin C; eGFR (CKD‐EPI), estimated glomerular filtration rate calculated using the Chronic Kidney Disease Epidemiology Collaboration equation, corrected for body surface area; GFR (DTPA clearance), radiolabeled diethylenetriaminepentaacetic acid clearance; Tc‐99 m‐DTPA, DTPA‐clearance.

The correlations of eGFR_CYS_, eGFR_CREA_, and eGFR_CYS‐CREA_ to mGFR measurements are shown in Table 3. Likewise, a comparison of eGFR_CYS_ and eGFR_CREA_ compared to mGFR is illustrated in Figure 1a,c. There was a strong correlation between the mGFR and eGFR_CYS_ (*R* ^2^ = 0.76, RMSE 12.62 mL/min/1.73m^2^) (Figure 1a), while the correlation between eGFR_CREA_ and mGFR was weaker (*R* ^2^ = 0.43, RMSE 18.88 mL/min/1.73m^2^) (Figure 1c). The correlation between eGFR_CREA‐CYS_ and mGFR was weaker than for eGFR_CYS_ alone (*R* ^2^ = 0.69) (not shown).

**TABLE 3:** Agreement between mGFR (DTPA clearance) (mL/min/1.73m^2^) and other parameters for all 248 participants.

	ICC (95% CI)
eGFR_CYS_ (mL/min/1.73m^2^)	0.92 (0.84–0.95)
eGFR_CREA_ (mL/min/1.73m^2^)	0.66 (0.011–0.84)
eGFR_CYS‐CREA_ (mL/min/1.73m^2^)	0.89 (0.83–0.93)

Abbreviations: crea, creatinine; cys: cystatin C; eGFR_(NN)_, estimated glomerular filtration rate calculated using the Chronic Kidney Disease Epidemiology Collaboration equation; ICC, intraclass correlation coefficient.

Differences between each of the eGFR methods and mGFR are plotted against the mGFR using Bland–Altman plots (Figure 1b,d). The mean difference for eGFR_CYS_ was −6.4 ± 12.7 mL/min/1.73m^2^, for eGFR_CREA_ 20.5 ± 20.3 mL/min/1.73m^2^ and for eGFR_CYS‐CREA_ 6.2 ± 14.3 mL/min/1.73m^2^ (not shown). Thus, eGFR_CREA_ greatly overestimated GFR, while eGFR_CYS_ only slightly underestimated GFR. The Bland–Altman analysis showed a better agreement for eGFR_CYS_ than for eGFR_CREA_.

In the fraction of participants with reduced relative renal function, differences between mGFR and eGFR_CYS_ were even smaller, with a bias of only 1.9 and similar confidence intervals.

Multivariable linear regression models for mGFR included age, gender, BMI, BSA, height, weight, s‐creatinine, s‐cystatin C, eGFR_CYS_, eGFR_CREA_, and eGFR_CYS‐CREA_. The most explanatory variable was the eGFR_CYS_, which accounted for 77% of the variance. The second most explanatory variable in the regression for the prediction of mGFR was weight, explaining together with eGFR_CYS_ 79% of the variance.

**FIGURE 2:**
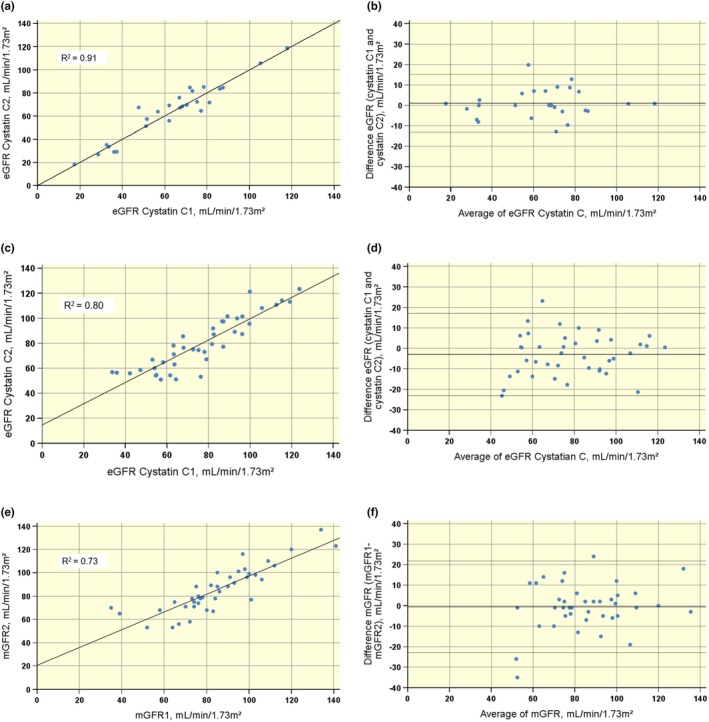
Test–retest variability (>1 month) of eGFR_CYS_ and long‐term repeatability (1–3.5 years) of eGFR_CYS_ and mGFR, with identity and Bland Altman plots. (a and b) Test–retest variability of eGFR_CYS_ in 26 participants (ICC 0.98 and CI 28.9 mL/min/1.73m^2^). (c and d) Long term repeatability of eGFR_CYS_ in 40 participants (ICC 0.94 and CI 41 mL/min/1.73m^2^) (C1 and C2: First and second measurement). (e and f) Long‐term repeatability of mGFR in 40 participants (ICC 0.92 and CI 45.6 mL/min/1.73m^2^), similar to that of eGFR_CYS_. CI, confidence interval; eGFR, estimated glomerular filtration rate calculated using the Chronic Kidney Disease Epidemiology Collaboration equation, corrected for body surface area; ICC, intraclass correlation coefficient; mGFR, radiolabeled diethylenetriaminepentaacetic acid clearance.

Test–retest variation of eGFR_CYS_, with an interval of measurements <3 months in 26 participants, showed very good repeatability (ICC: 0.98, CI: 28.9 mL/min/1.73m^2^, RMSE 7.37 mL/min/1.73m^2^, mean CV 0.06) (Figure 2a,b).

Long‐term repeatability (1–3.5 years) in 40 participants was similar and very good for eGFR_CYS_ (ICC: 0.94, CI: 41 mL/min/1.73m^2^, RMSE: 9.78 mL/min/1.73m^2^, mean CV: 0.09) (Figure 2c,d) and for mGFR (ICC 0.92, CI 45.6 mL/min/1.73m^2^, RMSE 10.3 mL/min/1.73 m^2^, mean CV 0.08) (Figure 2e,f).

**FIGURE 3:**
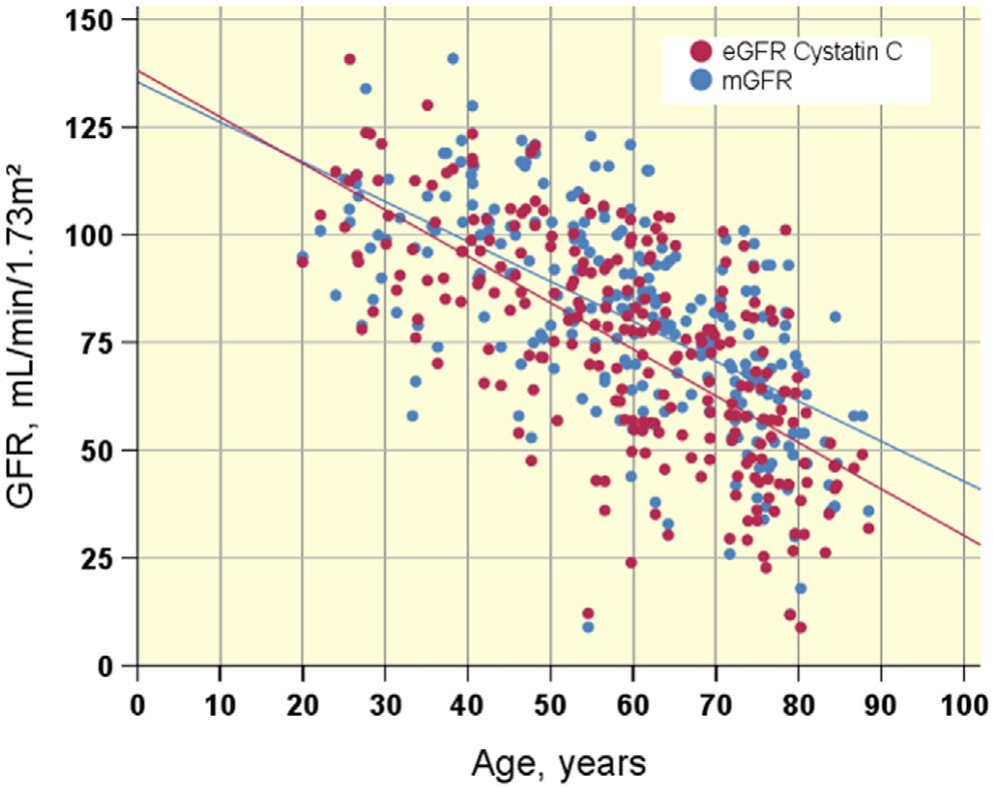
Plot of mGFR (DTPA clearance) and eGFR_CYS_ versus age in participants with spinal cord injury. The regression lines for mGFR and eGFR_CYS_ are approximately parallel and show an age‐related decline of renal function of 0.9 mL/year, which is within normal limits. Red dots: eGFR_CYS_. Blue dots: mGFR. eGFR cystatin C (CKD‐EPI), eGFR calculated using the Chronic Kidney Disease Epidemiology Collaboration equation; GFR, glomerular filtration rate; mGFR (DTPA clearance), radiolabeled diethylenetriaminepentaacetic acid clearance.

The age‐related decline of GFR in our population of 248 SCI individuals is 0.9 mL/min/year (Figure 3). Regression lines for mGFR and eGFR_CYS_ are almost parallel.

Eight subjects had a diagnosis of chronic kidney disease. This was the same eight subjects that were classified as having considerable or severely reduced kidney function according to the age‐ and gender‐dependent normal values for mGFR (Table 2).

We note that we use this classification when reporting mGFR to the clinicians.

**FIGURE 4:**
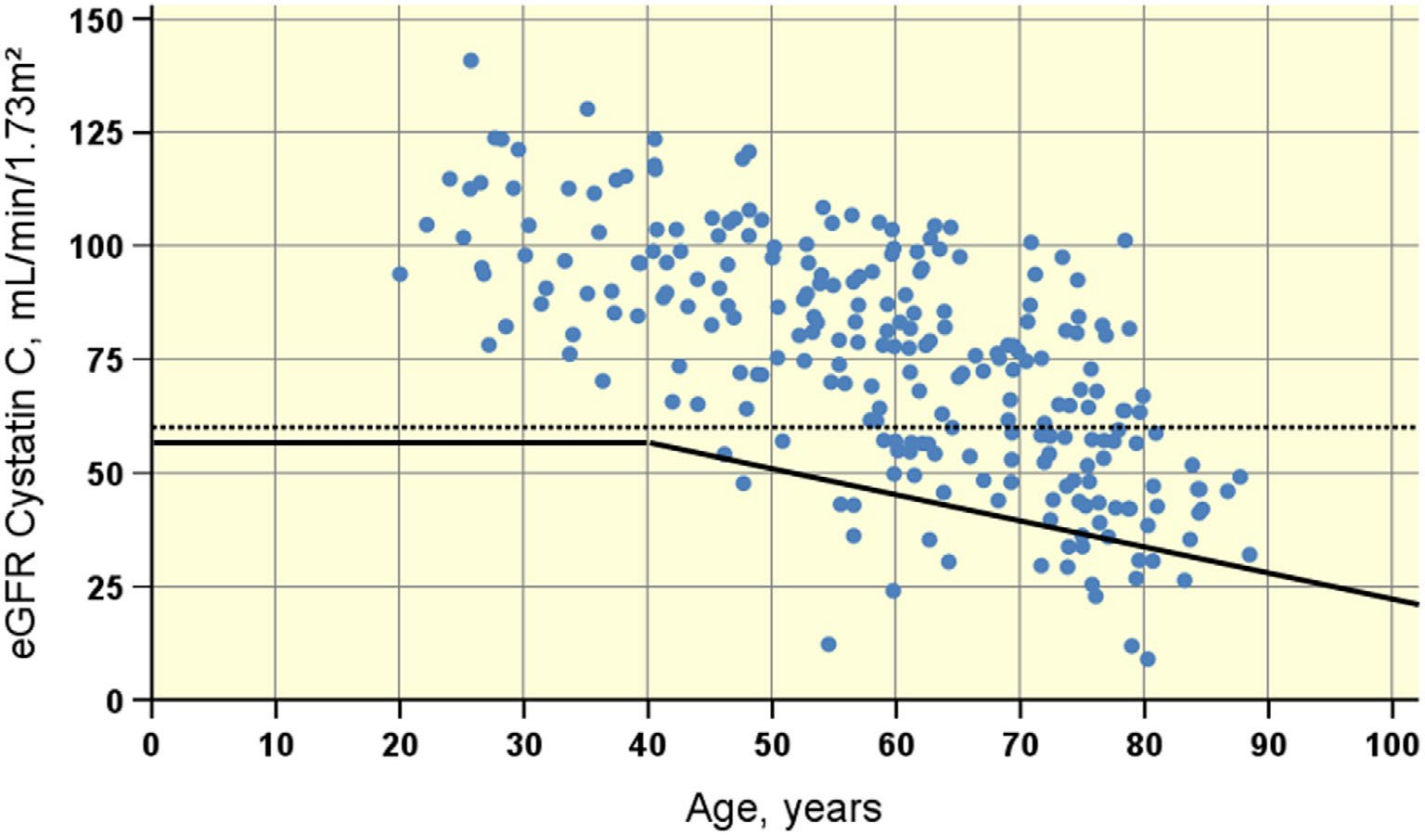
Plot of eGFR_CYS_ versus age in participants with spinal cord injury. The age‐corrected limit for considerably reduced renal function (Brochner‐Mortensen et al., 1977) is shown (solid line), together with the limit for Stage 3 Chronic Kidney Disease (NKF guidelines; dotted line). Using a limit not corrected for age will classify individuals <40 years similarly, but misclassify a significant number of individuals >40 years as having chronic kidney disease. eGFR cystatin C (CKD‐EPI), eGFR calculated using the chronic Kidney Disease Epidemiology Collaboration equation.

Figure 4 illustrates that using fixed limits for eGFR in the staging of chronic kidney disease, such as the Stage 3 CKD from KDIGO guidelines (Kidney Disease: Improving global outcomes (KDIGO) CKD work group, 2024) rather than the age‐corrected limit for considerably reduced renal function (Brochner‐Mortensen et al., 1977) will classify individuals <40 years similarly, but misclassify many individuals >40 years as having chronic kidney disease.

## 3 DISCUSSION

In this retrospective study, we compared eGFR calculated from a single measurement of s‐cystatin C using the CKD‐EPI formula (Inker et al., 2012, 2021; Levey et al., 2009), and eGFR from s‐creatinine, to the gold standard measured GFR using ^99m^Tc‐DTPA in individuals with neurogenic bladder dysfunction due to SCI. A close correlation between eGFR_CYS_ and mGFR was demonstrated and was equally good in participants with normal and reduced kidney function.

In contrast, the standard eGFR_CREA_ showed only a moderate correlation to the measured GFR, considerably overestimating the true clearance, particularly in participants with reduced kidney function. We thus confirm that the standard eGFR_CREA_ method (CKD‐EPI) based on measurements of s‐creatinine performs less well in these individuals with reduced muscle mass.

Using a combined equation with both s‐creatinine and s‐cystatin C was less accurate than with s‐cystatin C alone. In contrast, in individuals who do not have SCI, GFR calculated from a combination of s‐cystatin C and s‐creatinine levels appears to be advantageous. Thus, in a large validation data set from 12 studies including 4050 individuals over 18 years, it was found that an equation to estimate GFR, including both cystatin C and creatinine parameters, was more accurate for estimating kidney function than equations with either creatinine or cystatin C alone (Inker et al., 2021).

While this is the biggest study comparing eGFR calculated from s‐cystatin C and s‐creatinine to an external clearance method in individuals with SCI, many other studies have also compared these parameters, using either an isotope method or inulin clearance as reference clearance methods. The main results of these studies support the current data (Table 4) where the eGFR based on cystatin C is superior to the s‐creatinine‐based eGFR. *Erlandssen* et al. also analyzed the effect of having a complete versus an incomplete SCI, but found no significant differences. The authors found a good correlation to eGFR_CYS_ using either GFR from plasma and urine creatinine sampling or isotope clearance as mGFR. However, creatinine clearance with urine sampling is a more troublesome procedure than isotope clearance, particularly in individuals with SCI.

**TABLE 4:** Studies comparing eGFR using s‐cystatin C and s‐creatinine to an external clearance method in SCI individuals.

Authors, year	*N*	Clearance method	Correlation with mGFR
Current results	248	^99m^Tc‐DTPA	CC: eGFR_CREA_ (CKD‐EPI) = 0.66 eGFR_CYS_ = 0.76
Mingat er al, 2013	60	Inulin clearance	CC: eGFR_CREA_ (MDRD) = 0.57 eGFR_CYS_ = 0.80 Creatinine clearance = 0.72
Erlandsen et al., 2012	145	^51^Cr‐EDTA	AUC (ROC): eGFR_CREA_ (MDRD) = 0.71 eGFR_CYS_ = 0.83 Creatinine clearance = 0.82
Thomassen SA, 2002	24	^51^Cr‐EDTA	CC: 1/ cystatin C = 0.72 1/ creatinine = 0.26

Abbreviations: AUC (ROC), area under curve, receiver operating characteristics; CC, correlation coefficient; creatinine clearance, GFR from plasma and urine sampling of creatinine; eGFR_NN_ (CKD‐EPI), eGFR calculated using the EPI equation; eGFR_NN_ (MDRD), eGFR calculated using the MDRD equations.

No other study has specifically analyzed the correlations in individuals with reduced kidney function, which in our study is similar to participants with normal kidney function.

Monitoring of renal function is in routine clinical practice done by measurements of s‐creatinine or eGFR_CREA_. In special populations like individuals with SCI, it is recommended that mGFR should be performed at regular intervals, for example, 2 years (Ginsberg et al., 2021). It is currently debated whether reference clearance measurements at regular intervals can be substituted with eGFR_CYS_ measurements. Of note, GFR itself is not fixed but is subject to short‐term variations depending on food intake, hormonal changes, and other factors. In contrast, s‐cystatin C is not subject to short‐term changes, so while mGFR measures a mean of GFR in the determination period of 3 h, eGFR_CYS_ estimates a mean GFR over a much longer period. Nevertheless, the correlation between mGFR and eGFR_CYS_ in this study was very good, with ICC 0.92, considering that methodological variations in the s‐cystatin C determination as well as the mGFR determination are also included.

Substitution of the reference clearance measurements with eGFR_CYS_ measurements also requires knowledge of the repeatability of eGFR_CYS_. In other patient groups, variable coefficients of variation for cystatin C repeatability have been reported, ranging from excellent (Maahs et al., 2011) to poor (Olsson et al., 2010). Therefore, the test–retest variability of eGFR_CYS_ was assessed with repeated samples in 26 participants, taken at no more than 3‐month intervals (Figure 2a,b). Repeatability of these measurements was very good, with ICC 0.98.

The test–retest variability of ^99m^Tc‐DTPA clearance was not assessed. Previously, the test–retest variability of ^51^Cr‐EDTA clearance, which should be comparable to ^99m^DTPA clearance (Bröchner‐Mortensen & Rödbro, 1976; Vidal‐Petiot et al., 2021) has been investigated. In 51 individuals examined twice within 1 week, reproducibility was 5.1% (for GFR > 30 mL/min) and 11.5% (for GFR < 30 mL/min).

Long‐term repeatability of simultaneous eGFR_CYS_ and mGFR measures 1–3 years apart was good, with similar ICC for mGFR and eGFR_CYS_ ICC (Figure 2c,e and 2e,f).

The age‐related decline of GFR of 0.9 mL/min/year in our population with SCI compares well with the normal limits of age‐related decline of mGFR of 1 mL/min/year (Figure 3). eGFR_CYS_ and mGFR exhibit almost parallel declines with age. S‐cystatin C increases only slightly with age.

It is important to realize that even though there is a close correlation between eGFR_CYS_ and mGFR, and the two methods have a comparable long‐term repeatability that is classified as good, the confidence intervals for single measurements are so wide that neither method can reliably predict a change of 1 mL/min/year %. For the same reason, further statistics based on comparisons of the in‐subject variability of the two methods will be meaningless. On the other hand, it is clear from our data that the simple cystatin C‐based method is not inferior to the gold standard mGFR.

Overall, the current findings support that the much simpler eGFR_CYS_ should replace mGFR to estimate kidney function in individuals with SCI. From a clinical point of view, it is not trivial whether a tetraplegic patient in a wheelchair should have a blood sample taken every second year (e.g., at home) for determination of cystatin C, or come with special transportation to a Nuclear Medicine facility for a clearance measurement that lasts several hours and requires injection of a radioactive substance (even if the dose is small) and subsequent blood sampling.

The importance of using an age‐corrected normal material is demonstrated in Figure 4, where age is plotted against eGFR_CYS_. Comparing the age‐corrected limit for considerably reduced renal function (Brochner‐Mortensen et al., 1977) to the limit for stage 3 CKD from KDIGO guidelines (Kidney Disease: Improving global outcomes (KDIGO) CKD work group, 2024) shows that using a limit not corrected for age will classify individuals <40 years similarly, but misclassify a significant number of individuals >40 years as having chronic kidney disease. The consequence of using fixed limits of GFR (e.g., 60 mL/min to denote definite CKD) will be that many old people with age‐appropriate GFR will be falsely classified as having CKD and associated higher cardiovascular risk. The arguments against using fixed limits have been discussed recently by Delanaye et al. (2012). For s‐cystatin C, similar misclassifications will occur if a normal material with a fixed normal limit is used.

### 3.1 Perspectives

In other patient groups such as children, anorectic individuals, and elderly individuals with low muscle mass, eGFR_CREAT_ is less exact for estimating the true GFR. For these groups, eGFR_CYS_ will likely perform better in these circumstances, and studies should be undertaken to verify this.

### 3.2 Limitations

Limitations of this study may be the retrospective design and a relatively low number of individuals (20.1%) with reduced renal function. However, it is an unselected sample of individuals with SCI, referred from the Department of Spinal Cord Injuries for their standard follow‐up of kidney function.

## 4 CONCLUSIONS

We find a very good correlation between the measured DTPA clearance (mGFR) and the estimated clearance calculated from cystatin C (eGFR_CYS_) in individuals with SCI, with an almost parallel age‐related decline. The agreement is equally good in individuals with normal and reduced kidney function. In contrast, eGFR_CREA_ shows a poor correlation to mGFR.

The test–retest variation of eGFR_CYS_ measurements in individuals with SCI is very good, and the long‐term (1–3 years) repeatability of eGFR_CYS_ and mGFR is very good and comparable. This indicates that eGFR_CYS_ should replace the more time‐consuming and labor‐intensive mGFR measurements for monitoring kidney function in individuals with SCI. We further recommend to use eGFR with age‐corrected normal limits rather than s‐cystatin C or eGFR_CYS_ with fixed limits,as this will misclassify a substantial number of particularly older people as having decreased renal function.

## 5 MATERIALS AND METHODS

### 5.1 Study design

This study included a consecutive and unselected sample of 248 individuals with chronic SCI, referred from the Department of Spinal Cord Injuries, Copenhagen University Hospital, Rigshospitalet, Glostrup for the normal follow‐up controls of their kidney function, performed during the period June 2020–January 2022.

Participants provided written informed consent to collect an extra blood sample for s‐cystatin C and s‐creatinine determination alongside their regular ^99m^Tc‐DTPA clearance examination.

The study was performed at the Department of Clinical Physiology and Nuclear Medicine, Copenhagen University Hospital, Rigshospitalet, Glostrup as a quality assurance study and was approved by the local research committee and Legal entity of Copenhagen University Hospital, Rigshospitalet (approval number p‐2024‐17,009). The study was conducted according to the principles of the Declaration of Helsinki.

### 5.2 Methods

^99m^Tc‐DTPA clearance (mGFR) was measured at the Department of Clinical Physiology and Nuclear Medicine, Rigshospitalet, Glostrup. The single‐plasma sample, single‐tracer injection ^99m^Tc method was used (Groth & Aasted, 1981).

Eight MBq ^99m^Tc‐DTPA (TechneScan DTPA®, Curium Netherlands) was injected intravenously, and two venous blood samples were collected from the arm after 200 min. Plasma radioactivity was determined in a Wizard 2740 gamma well counter (PerkinElmer, Waltham, MA, USA). GFR was calculated using the equation presented by Groth and Aasted (1981). The height and weight of the participants were recorded.

Blood samples were also drawn for analysis of s‐cystatin C and s‐creatinine levels. S‐cystatin C was analyzed using a Cobas, CYSC2 Tina‐quatt Cystatin C generation 2 (Roche Diagnostics Ltd., Rotkreuz, Switzerland) (Erlandsen & Randers, 2010; Hansson et al., 2010). S‐creatinine was analyzed using the VITROS Chemistry Products CREA Slides REF 6802584 (Ortho Clinical Diagnostics, Inc., Rochester, USA).

The laboratory reference interval was 0.61–0.95 mg/L for s‐cystatin C. The reference intervals for s‐creatinine were 0.68–1.19 mg/dL for men and 0.57–1.02 mg/dL for women.

The estimated GFR was calculated using the s‐cystatin C‐based (eGFR_CYS_), creatinine‐based (eGFR_CREA_), and combined s‐cystatin C‐creatinine‐based eGFR (eGFR_CYS‐CREA_) using CKD‐EPI equations (Inker et al., 2012, 2021; Levey et al., 2009). CKD‐EPI equations incorporating factors such as age and gender, in addition to the values of s‐creatinine or s‐cystatin C, were used as they are more accurate than the Modification of Diet in Renal Disease (MDRD) Study equation (Levey et al., 1999, 2006) across a wide variety of populations and clinical conditions.

The eGFR values eGFR_CYS_, eGFR_CREA_, and eGFR_CYS‐CREA_ as well as mGFR values were adjusted to a body surface area of 1.73m^2^ using the equation by Du Bois and Du Bois (1989).

The population was stratified into four degrees of relative renal function, with the delimitations expressed as a percent of the corresponding age‐ and gender‐dependent normal mean GFR (Brochner‐Mortensen et al., 1977):

1. Normal (>75%)
2. Moderately decreased (75%–52%)
3. Considerably decreased (51%–28%)
4. Severely decreased (<28%).

### 5.3 Statistical analysis

All statistical analyses were performed using IBM SPSS Statistics 28. Data are presented as group mean ± standard deviation (SD) unless otherwise stated. All analyses were performed both for all participants and separately for women and men. Gender differences were evaluated using Student's *t*‐test for independent samples and Mann–Whitney U tests for two independent groups. In addition, the association between age, weight, height, and measured variables was assessed by regression analyses. Least square linear and quadratic regression models were compared based on the coefficient of determination (*R* ^2^) to determine the most appropriate regression model.

Pearson correlations were performed to examine the relationship between mGFR and the various eGFR. Agreement between the methods was assessed using the Bland–Altman method (Bland & Altman, 1999) and reliability employing the IntraClass Correlation Coefficient (ICC). When evaluating ICC, the closer the coefficient is to 1, the higher the reliability. We considered an ICC over 0.90 as very high, between 0.70 and 0.89 as high, and between 0.50 and 0.69 as moderate. For each linear regression, the Root mean square error (RMSE) was also reported.

For the analysis of variance, two repeated measures of s‐cystatin C from a subgroup of 26 participants taken within 3 months of each other were analyzed using ICC. Residuals were checked for normal distribution by one‐sample Kolmogorov–Smirnov test and visual inspection. An analysis of repeated measures over a longer period was performed in 40 individuals to compare the variance of both eGFR and mGFR measures using ICC, and a regression was performed to identify an eventual age‐related decline in mGFR/eGFR for the studied SCI population.

Multiple regression analyses (stepwise) were used to explore potential contributions of the parameters: age, gender, BMI, BSA, height, weight, s‐creatinine, s‐cystatin C, eGFR_CYS_, eGFR_CREA_, and eGFR_CYS‐CREA_. Differences were considered significant at *p* < 0.05.

## FUNDING INFORMATION

The study received no external funding.

## CONFLICT OF INTEREST STATEMENT

FBS reports personal consulting fees from Neuroplast—The Netherlands; Coloplast—Denmark; Sunnaas Rehabilitation Hospital – Norway. CK reports institutional grants from the Danish Cancer Society; the Novo Nordisk Foundation; Local PI on multicenter study from Bayer (Oceanic) on stroke treatment: Local PI on a study from Coloplast on urinary sedimentation in spinal cord injury patients. All other authors have nothing to declare.

## ETHICS STATEMENT

The study was performed at the Department of Clinical Physiology and Nuclear Medicine, Copenhagen University Hospital, Rigshospitalet as a quality assurance study and was approved by the local research committee and Legal entity of Copenhagen University Hospital, Rigshospitalet (approval number p‐2024‐17,009). The study was conducted according to the principles of the Declaration of Helsinki.

## PATIENT CONSENT STATEMENT

Participants provided written informed consent to collect an extra blood sample for s‐cystatin C and s‐creatinine determination alongside their regular ^99m^Tc‐DTPA clearance examination.

## CLINICAL TRIAL REGISTRATION

The study is registered at ClinicalTrials.gov (ClinicalTrials.gov ID: NCT06565351).

## SOCIAL MEDIA AND PROMOTION TEXT

In individuals with spinal cord injury (SCI) kidney function (GFR) is regularly monitored by injection of, for example,^99m^DTPA and subsequent blood sampling, a tedious procedure. This study compared eGFR calculated from a single plasma sample of the endogenous substance Cystatin C to ^99m^DTPA‐GFR in 248 individuals with SCI. We conclude that eGFR based on a single sample of plasma cystatin C can replace the more time‐consuming and labor‐intensive ^99m^DTPA‐GFR procedures in individuals with SCI.

## Data Availability

The data that support the findings of this study are available from the corresponding author, UBA, upon reasonable request.
